# Evolution of Abscisic Acid Signaling for Stress Responses to Toxic Metals and Metalloids

**DOI:** 10.3389/fpls.2020.00909

**Published:** 2020-07-17

**Authors:** Beibei Hu, Fenglin Deng, Guang Chen, Xuan Chen, Wei Gao, Lu Long, Jixing Xia, Zhong-Hua Chen

**Affiliations:** ^1^Engineering Research Center of Ecology and Agricultural Use of Wetland, Ministry of Education/Hubei Key Laboratory of Waterlogging Disaster and Agricultural Use of Wetland, Yangtze University, Jingzhou, China; ^2^State Key Laboratory of Crop Stress Adaptation and Improvement, Henan University, Kaifeng, China; ^3^State Key Laboratory for Conservation and Utilization of Subtropical Agro-bioresources, College of Life Science and Technology, Guangxi University, Nanning, China; ^4^School of Science, Western Sydney University, Penrith, NSW, Australia; ^5^Hawkesbury Institute for the Environment, Western Sydney University, Penrith, NSW, Australia

**Keywords:** plant evolution, comparative genomics, cadmium, arsenic, lead, detoxification

## Abstract

Toxic heavy metals and metalloids in agricultural ecosystems are crucial factors that limit global crop productivity and food safety. Industrial toxic heavy metals and metalloids such as cadmium, lead, and arsenic have contaminated large areas of arable land in the world and their accumulation in the edible parts of crops is causing serious health risks to humans and animals. Plants have co-evolved with various concentrations of these toxic metals and metalloids in soil and water. Some green plant species have significant innovations in key genes for the adaptation of abiotic stress tolerance pathways that are able to tolerate heavy metals and metalloids. Increasing evidence has demonstrated that phytohormone abscisic acid (ABA) plays a vital role in the alleviation of heavy metal and metalloid stresses in plants. Here, we trace the evolutionary origins of the key gene families connecting ABA signaling with tolerance to heavy metals and metalloids in green plants. We also summarize the molecular and physiological aspects of ABA in the uptake, root-to-shoot translocation, chelation, sequestration, reutilization, and accumulation of key heavy metals and metalloids in plants. The molecular evolution and interaction between the ABA signaling pathway and mechanisms for heavy metal and metalloid tolerance are highlighted in this review. Therefore, we propose that it is promising to manipulate ABA signaling in plant tissues to reduce the uptake and accumulation of toxic heavy metals and metalloids in crops through the application of ABA-producing bacteria or ABA analogues. This may lead to improvements in tolerance of major crops to heavy metals and metalloids.

## Introduction

Toxic mineral elements, such as metals and metalloids, are ubiquitous in the Earth's crust. The amount of toxic metals and metalloids in water and soil is increasingly attributed to anthropogenic activities (Bowell et al., 2014; Zhu et al., 2014; Zhao et al., 2015). These contaminates affect agricultural productivity and ecosystem function and also threat human health, posing a great risk to global economic growth (Landrigan et al., 2018). Moreover, toxic metals and metalloids accumulated in edible parts of plants, such as cereals and vegetables, as well as in pasture for animals, should be tightly controlled to reduce health risks. Therefore, urgent actions are required to mitigate the serious problems from heavy metals and metalloids' contamination.

Since the evolution of land plants from ancestral green algae (Cheng et al., 2019; Zhao et al., 2019; Wang et al., 2020), land plants have been indispensable to the biosphere and our daily life. In addition to feeding the world's population, many plant species have also been employed to alleviate the increasing pollution of heavy metals and metalloids through bioremediation. Over 700 plant species have been identified as hyperaccumulators of trace metals, metalloids, and nonmetals. The hyperaccumulator species are from 52 families belonging to angiosperms and petridophyta (Reeves et al., 2017). For instance, one plant used is a hardy, versatile, fast-growing brake fern (*Pteris vittata*) with extreme efficiency in extracting and translocating soil arsenic (As) to the above-ground biomass, which can significantly remove arsenic from contaminated soils (Ma et al., 2001; Yan et al., 2019). Therefore, exploring the early-divergent plant species for their tolerance mechanisms is essential to utilize them as potential hyperaccumulators for heavy metals and metalloids.

Among the toxic minerals, Arsenic, cadmium (Cd), and lead (Pb) were ranked as the top hazardous substances (Clemens and Ma, 2016) due to their toxicity, prevalence, and potential for human exposure. During the last decade, considerable advances in As and Cd accumulation and detoxification mechanisms in angiosperms, in particular the model plant species rice (*Oryza sativa*) and Arabidopsis (*Arabidopsis thaliana*) (Clemens and Ma, 2016; Singh et al., 2016; Deng et al., 2019; Deng et al., 2020; Zhao and Wang, 2020) have been made. Although genetic engineering is a powerful strategy for generating ideal plants for food safety and phytoremediation (Shim et al., 2013; Deng et al., 2018), it's not widely used in agriculture at present due to its controversy in potential risk to human health and agricultural ecosystems (Andersen et al., 2015). Therefore, understanding the molecular mechanisms of elements absorption and root-to-shoot translocation make it possible to promote the efficiency of phytoremediation instead of genetic engineering.

Abscisic acid (ABA) plays vital roles in plant responses to a range of abiotic stresses such as drought, salinity, high light, nutrient deficiency, and heavy metals (Chen Z. H. et al., 2016; Cai et al., 2017; Liu X. et al., 2017; Vishwakarma et al., 2017; Wang F. et al., 2017; Kuromori et al., 2018; Babla et al., 2019; Mak et al., 2019; Mega et al., 2019; Feng et al., 2020; Shabala et al., 2020) and its agonist and antagonist were considered as candidate compounds to overcome these stresses (Joshi-Saha et al., 2011; Kitahata and Asami, 2011; Miyakawa et al., 2013; Park et al., 2015; Gupta et al., 2020). ABA is one of the foremost phytohormone driving plant resistance to toxic metals and metalloids such as As, Cd, and Pb (Maestri and Marmiroli, 2012; Chmielowska-Bak et al., 2014; Vishwakarma et al., 2017; Amir et al., 2018; Pál et al., 2018; Shi et al., 2019; Zhang P. et al., 2019; Zhang W. et al., 2019; Pan et al., 2020). Mechanisms of ABA in response to heavy metals and metalloids stresses in non-angiosperm plant lineages is still limited; we took a comparative genomic evolutionary approach to shed some light on the insights of ABA and tolerance to heavy metals and metalloids.

There have been many excellent reviews on ABA and plant stress tolerance in recent years (Osakabe et al., 2014; Mittler and Blumwald, 2015; Zhu, 2016; Hauser et al., 2017; Kuromori et al., 2018; Chen et al., 2020). Here, we summarize three ABA-activated pathways that contribute to heavy metals detoxification in angiosperms using rice and Arabidopsis as model species. We also attempt to trace the origin and evolution of the core components linking ABA and tolerance to toxic metals and metalloids involved in the processes.

## Overview of ABA Signaling Network

Abscisic acid (ABA) is a vital phytohormone that regulates many developmental processes in plants and in the response to abiotic stresses including drought, cold, salinity, and heavy metals (Chmielowska-Bak et al., 2014; Mittler and Blumwald, 2015; Chen et al., 2017; Hauser et al., 2017; Chen et al., 2019; Zhang P. et al., 2019; Zhao et al., 2019). The biosynthesis, catabolism, transport, signal perception and transduction, downstream response, and modulation of ABA have been extensively investigated in angiosperms, in particular in *Arabidopsis thaliana* (Hauser et al., 2011; Cai et al., 2017; Hauser et al., 2017; Chen et al., 2020).

ABA is primarily synthesized from carotenoids, which are catalyzed by various enzymes including β-carotene hydroxylases, zeaxanthin epoxidase (ZEP, ABA1), 9-cis-epoxycarotenoid dioxygenase (NCEDs), short-chain alcohol dehydrogenase/reductases (SDRs, such as ABA2), abscisic aldehyde oxidases (AAOs), molybdenum cofactor sulfurase (MOCO, ABA3), and ABA4, which is required for neoxanthin synthesis (Nambara and Marion-Poll, 2005; North et al., 2007; Finkelstein, 2013; Cai et al., 2017; Hauser et al., 2017). The hydroxylation and esterification of ABA are two major pathways for regulating ABA levels mediated by four CYP707As and eight glucosyltransferases (UGTs). The inactivated ABA-glucosyl ester (ABA-GE) conjugation is a storage or transport form of ABA and the site can be cleaved by β-glucosidases (BGLUs) (Finkelstein, 2013). The mobility of ABA from vascular tissues to target tissues is transported by three groups of membrane-localized proteins: G-type ATP binding cassette transporters (ABCG22, ABCG25, ABCG30, ABCG31, ABCG40), ABA-Importing Transporters (AIT1~4) belonging to the Nitrate Transporter 1/Peptide Transporter (NRT1/PTR, NPF) family, and a member of DTX/Multidrug and Toxic Compound Extrusion (MATE) family DTX50 in Arabidopsis (Kang et al., 2010; Kuromori et al., 2010; Kuromori et al., 2011; Kanno et al., 2012; Zhang et al., 2014; Kang et al., 2015).

The core components of ABA perception and transduction consist of intracellular Pyrabactin Resistance 1 (PYR1)/PYR-Like (PYL)/Regulatory Component of ABA receptors (RCARs), clade A Protein Phosphatases PP2C (ABA Insensitive 1/2, ABI1/2, and Hypersensitive to ABA1/2, HAB1/2), sucrose non-fermenting-1-related protein kinase 2 family members (SnRK2s) (Ma et al., 2009; Park et al., 2009; Umezawa et al., 2009). SnRK2.2, -2.3, and -2.6 (Open Stomata 1, OST1) are strongly activated in the presence of ABA (McLoughlin et al., 2012). SnRK2.6 is a key regulator of stomatal closure by enhancing Cl^-^, K^+^, malate^2-^ efflux from guard cells mediated by S-type anion channel 1 (SLAC1), K^+^ uptake transporter 6 (KUP6), and Aluminum-activated malate transporter (ALMT12), and inhibiting the activity of Potassium channel 1 (KAT1) to reduce K^+^ influx (Finkelstein, 2013; Chen et al., 2020). ABA also enables the activation of guard cell membrane-localized transporters through phosphorylation mediated by calcium dependent kinases (CDPKs) and other kinases, indicating alternative stomatal regulatory pathways independent of SnRK2s (Cai et al., 2017; Pornsiriwong et al., 2017). Most recently, subgroup B Raf-like kinases have been identified as upstream regulators of SnRK2s for ABA signal transduction and response to osmotic and drought stresses in Arabidopsis (Saruhashi et al., 2015; Katsuta et al., 2020; Lin et al., 2020; Soma et al., 2020; Takahashi et al., 2020). Furthermore, it's revealed that this regulatory module is evolutionarily conserved in land plants, at least for conferring protection against drought (Katsuta et al., 2020). The other biological regulations induced by ABA are mostly implemented through transcriptional processes mediated by ABA Insensitive 3/4 (ABI3/4) and 9 ABA-response element (ABRE) binding factors (ABFs) (Chen et al., 2020). *ABI3* and *ABI4* genes encode B3-type and APETALA2 domain a transcription factors, respectively, while ABFs consisting of ABF1~4, ABI5, bZIP15, bZIP67, and EEL (Enhanced Em Level) belong to group A subfamily of bZIP (basic region/leucine zipper) transcription factors (Choi et al., 2000; Finkelstein, 2013; Huang et al., 2016; Skubacz et al., 2016; Chen et al., 2020). All the listed components are candidates for the role of ABA in response to toxic metals and metalloids stresses.

## ABA Alleviates Toxic Metals and Metalloids Stresses in Plants

First of all, the biosynthesis and signaling pathways of ABA are affected by heavy metal stresses. Elevated endogenous ABA content was detected in rice, potato (*Solanum tuberosum*), oilseed rape (*Brassica napus*), *Malus hupehensis*, *Sedum alfredii*, and other plants exposed to Cd, partially due to the upregulation of genes for ABA biosynthesis (Hsu and Kao, 2003; Stroiński et al., 2013; Yan et al., 2016; Shi et al., 2019; Zhang W. et al., 2019; Lu et al., 2020b). In rice, the expression levels of *OsNCED3*, *OsNCED4*, and *OsNCED5* were upregulated by Cd (Tan et al., 2017). In addition, Cd-induced rapid ABA production was more significant in the leaves and roots of Cd-tolerant rice cultivar than those in the Cd-sensitive genotype (Hsu and Kao, 2003), indicating the positive correlation between endogenous ABA content and Cd tolerance.

In Arabidopsis, enhanced Cd sensitivity and increased Cd accumulation was observed in three ABA-deficient mutants (*aba-1*, *aba-3*, *aba-4, nced3*) and two ABA-insensitive mutants (*abi2-1, abi3-1*) (Sharma and Kumar, 2002; Zhang W. et al., 2019). These genes are involved in ABA synthesis (*ABA1*, *ABA3*, *ABA4, nced3*) and signal transduction (*ABI2*, *ABI3*), respectively. Similarly, the Arabidopsis mutants *bglu10* and *bglu18* with reduced root cytoplasmic ABA levels were more sensitive to Cd stress compared to the wild type (**Table 1**) (Wang et al., 2018). Ectopic expression of *Malus hupehensis NCED3* (*MhNCED3*) in Arabidopsis increased ABA content and reduced Cd accumulation in both root and leaves (Zhang W. et al., 2019). Moreover, in Cd-contaminated soil, greater biomass and lower Cd concentrations were determined in Arabidopsis and *Brassica chinensis* inoculated with ABA-generating bacteria, *Azospirillum brasilense* or *Bacillus subtilis* (Xu et al., 2018; Pan et al., 2019). By contrast, decreasing the endogenous ABA amount by inoculation with an ABA-catabolizing bacteria, *Rhodococcus qingshengii*, significantly increased Cd content in Arabidopsis shoots by 47% (Lu et al., 2020a). Compared to the wild type, higher root Cd concentration was detected in ABA-deficient *Slsit* tomato mutant (**Table 1**) (Pompeu et al., 2017). Application of ABA further enhanced Cd tolerance and accumulation activity in the Cd-hyperaccumulating ecotype (HE) of *Sedum alfredii* (Lu et al., 2020b). Interestingly, elevated levels of endogenous ABA accompanied by up-regulated *SaNCED* and *SaABA2* was observed in the non-hyperaccumulating ecotype (NHE) subjected to Cd treatment compared to those in HE, restricting radial transport of Cd toward root vascular tissues (Tao et al., 2019; Lu et al., 2020b).

**Table 1:** Phenotype of mutant lines under Cd treatment.

Species	Genotypes	ABA condition	Physiological performace compared to control (wild type)	References
*Arabidopsis thaliana*	*abi5-1*	ABA-insensitive	Increased Cd accumulation and reduced root elongation but not affacted by ABA	(Zhang P. et al., 2019)
	*myb49-1/2, MYB49-SRDX*	Normal	Reduced Cd accumulation, enhanced Cd tolerance but recovered by overexpressing *HIPP44*	
	*ait1*	ABA transporter defective	Increased Cd in roots and leaves and reduced Cd tolerance but partially abolished by ABA	(Fan et al., 2014; Xu et al., 2018; Lu et al., 2019; Pan et al., 2020)
	*irt1*	Normal	Reduced Cd in roots and shoots but not largely afffacted by ABA	
	*snrk2.2/2.3*	ABA-insensitive	ABA-inhibited Cd accumulation was abolished	
	*abi1/abi2/hab1*	ABA-sensitive	Promoted Cd accumulation by inoculation with ABA-catabolizing bacteria	(Lu et al., 2019)
	*bglu10/18*	Reduced active form of ABA	Enhanced Cd sentivity	(Wang et al., 2018)
	*aba-1*	Normal	Inhibited Cd sensitivity	(Sharma and Kumar, 2002)
	*aba-3*	ABA-deficient		
	*aba-4*	ABA-deficient		
	*abi2-1*	ABA-insensitive		
	*abi3-1*	ABA-insensitive		
	*nced3*	ABA-deficient	Enhanced Cd accumulation in roots and leaves	(Zhang W. et al., 2019)
Tomato (*Solanum* *lycopersicum*)	*Slsit*	ABA-deficient	Higher Cd concentration in the roots	(Pompeu et al., 2017)

Elevated ABA content was increased in both roots and shoots of As-exposed Indian mustard (*Brassica juncea*), which is a potential As accumulator for phytoremediation (Srivastava et al., 2013). Furthermore, ABA-related genes were regulated predominately in As-tolerant ecotype Col-0, however, all the 25 genes involved in ABA biosynthesis, receptor, and signaling pathways detected in sensitive ecotype Ws-2 were unaltered by the treatment of As (Fu et al., 2014). In addition to the upregulated *OsNCED1*, *OsNCED2*, *OsNCED3*, and *OsABA4* responsible for ABA biosynthesis, the expression levels of genes probably involved in ABA signaling including *OsPP2Cs*, *OsbZIP10* (*OsABI5*)*, OsbZIP12* (*OsABF1*)*, OsbZIP66* (*OsABF5*), and *OsbZIP72* (*OsABF4*) were also elevated when rice plants were exposed to As (Huang et al., 2012; Yu et al., 2012). Moreover, increased endogenous ABA levels were detected in germinating chickpea (*Cicer arietinum*) and leaves of pea (*Pisum sativum*) exposed to Pb (Parys et al., 1998; Atici et al., 2005). Compared to that of control, ABA concentration was increased by 107% in the leaves of Gray Poplar (*Populus × canescens*) with Pb exposure, while application of exogenous ABA alleviated Pb toxicity (Shi et al., 2019). Recently, comparative transcriptomic analyses between Arabidopsis (Pb sensitive) and *Hirschfeldia incana* (Pb tolerant) revealed that genes involved in ABA biosynthesis were upregulated in the roots and shoots of *H. incana* subjected to Pb (Auguy et al., 2016).

**Figure 1:**
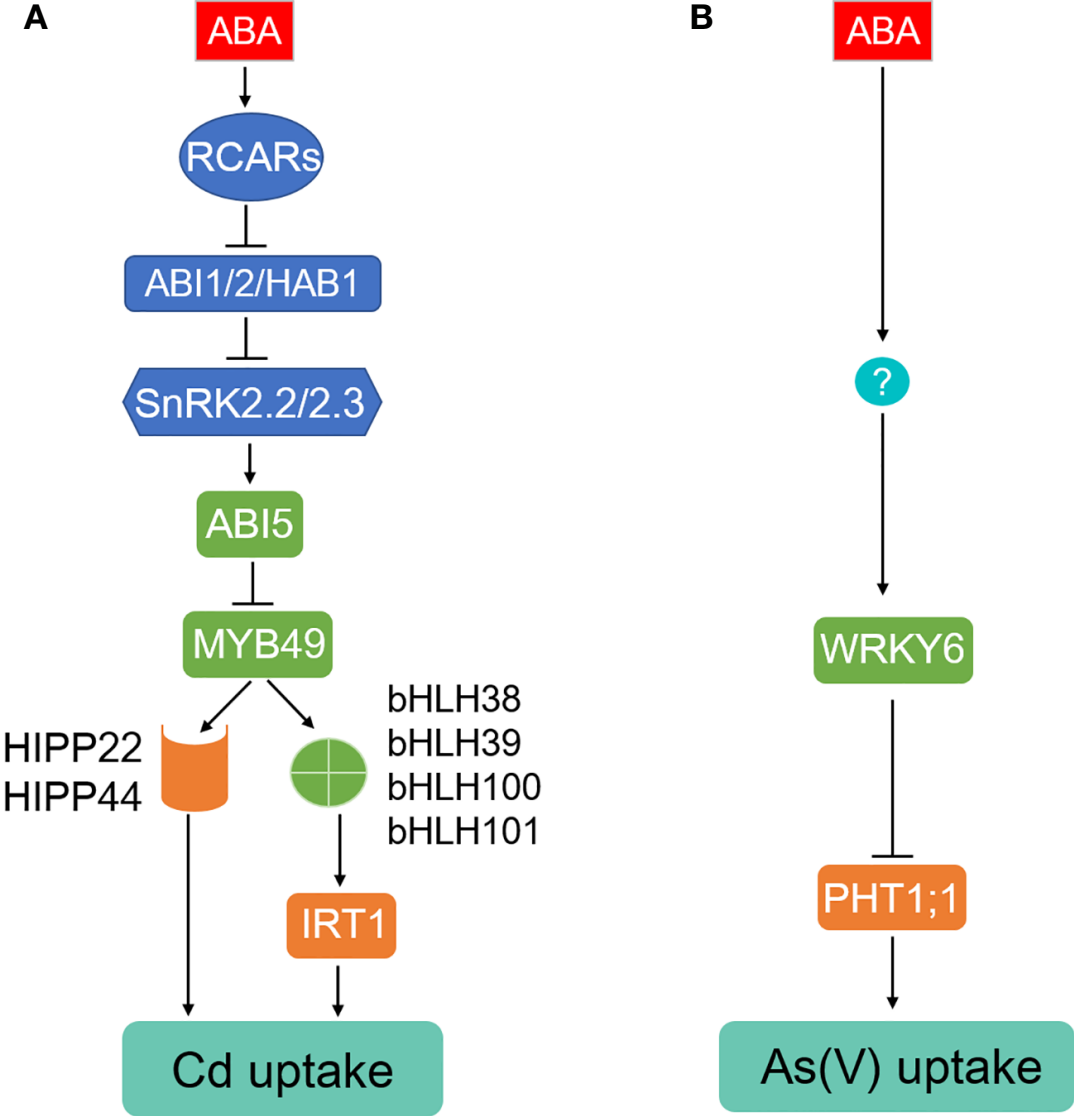
ABA inhibits Cd and As(V) uptake in Arabidopsis. **(A)** Cd uptake and accumulation mediated by IRT1 and HIPP proteins is activated by a transcriptional factor MYB49, which can be inactivated by interaction with ABA-induced ABI5. **(B)** Arsenate uptake mediated by PHT1;1 is repressed by ABA-induced transcriptional factor WRKY6.

These imply a positive role of ABA in alleviating accumulation and toxicity of heavy metals and metalloids. Consistently, the application of exogenous ABA to angiosperms subjected to Cd, As, or Pb could alleviate the stresses. Three major pathways involved in the detoxification of toxic metals and metalloids can be triggered by ABA, inhibiting the uptake, altering the translocation from root to shoot, and promoting the conjugation with chelators (**Figure 1**).

## ABA Limits Toxic Heavy Metals and Metalloids Uptake

In Arabidopsis, Iron Regulated Transporter 1 (IRT1) functions as a primary transporter for Cd uptake from the rhizosphere (Lux et al., 2011). The expression of *IRT1* is tightly regulated by FIT (FER-like Deficiency Induced Transcripition Factor) and Ib subgroup of the bHLH (basic helix-loop-helix) transcriptional factors including bHLH38, bHLH39, bHLH100, and bHLH101 in Arabidopsis (Wu et al., 2012; Wang et al., 2013). Application of low concentrations of ABA (0.1~1.0 μM) or inoculation with ABA-generating bacteria strains decreased Cd uptake through inhibiting transcription of *IRT1* and thereby alleviating Cd-induced growth inhibition (Fan et al., 2014; Zhang P. et al., 2019; Pan et al., 2020). By contrast, an increase was observed in expression level of *IRT1* and its homologous genes such as *ZIP1* (*Zinc Regulated Transporter/IRT-like Protein 1*) and *ZIP4* with Cd transporting-activity in the roots inoculated with ABA-catabolizing bacteria (Xu et al., 2018; Pan et al., 2019; Lu et al., 2020a). These positive effects were reduced in the ABA-Importing Transporter 1 (AIT1)-deficient mutant *ait1* but enhanced in the *AIT1*-overexpressing transgenic plants (Pan et al., 2020). Furthermore, the loss-of-function Arabidopsis mutant of ABI5 and ABA-insensitive double mutant of *snrk2.2/2.3* abolished the reduction of Cd accumulation induced by exogenously applied ABA or ABA-generating bacteria. The elevated *IRT1* transcription was diminished in *snrk2.2/2.3* mutant but further enhanced in the roots of ABA-sensitive triple PP2Cs mutant *abi1/hab1/abi2* lines, suggesting the involvement of AIT1, ABI5, SnRK2s, and PP2Cs in Cd absorption mediated by IRT1 (Fan et al., 2014; Xu et al., 2018; Pan et al., 2019; Zhang P. et al., 2019; Lu et al., 2020a). Additionally, ABI5 can be directly phosphorylated by SnRK2.2/2.3 and inactivated *via* dephosphorylation of ABI1/HAB1/ABI2 (Fujii et al., 2009; Skubacz et al., 2016). ABI5 could directly interact with Cd-induced transcriptional factor MYB49 to inhibit its binding to the promoter regions of bHLH38 and bHLH101, which are required for *IRT1* expression (Wu et al., 2012; Wang et al., 2013; Zhang P. et al., 2019). As a result, ABI5 is a negative regulator of IRT1 and Cd uptake. Moreover, MYB49 directly induces the expression of *HIPP22* and *HIPP44*, members belonging to Heavy metal-associated Isoprenylated Plant Proteins family (HIPPs) involved in Cd accumulation (Tehseen et al., 2010; Zhang P. et al., 2019). The expression of Arabidopsis *HIPP22* and *HIPP44* are positively correlated with Cd accumulation (Tehseen et al., 2010; Zhang P. et al., 2019). It is noteworthy that tobacco (*Nicotiana tabacum*) and Arabidopsis SnRK2s are transiently activated by Cd exposure, knockout of *AtSnRK2.4* enhanced Cd tolerance. However, the induced *SnRK2.4* is ABA-independent but probably influences ROS accumulation (Kulik et al., 2012).

Cd absorption was also observed to be inhibited in lettuce (*Lactuca sativa*) and oilseed rape (*Brassica napus*), however, whether this is a conserved route is not clear (Shen et al., 2017; Tang et al., 2020). On the other hand, Cd absorption was not affected by ABA in rice, Indian mustard, and *A. helleri* (Salt et al., 1995; Hsu and Kao, 2003; Zhao et al., 2006), which could be due to a different Cd uptake system in these species. For instance, the major transporters for Cd uptake in rice is OsNramp5 (Sasaki et al., 2012) with less contribution from OsIRT1 and OsIRT2 (Nakanishi et al., 2006). OsNramp5 is a member belonging to Natural Resistance-Associated Macrophage Protein family and polarly localized at the distal side of both root exodermis and endodermis cells (Sasaki et al., 2012). Knockout of *OsNramp5* almost abolished the ability to take up Cd (Ishikawa et al., 2012; Sasaki et al., 2012). In summary, we propose that ABA alleviates Cd uptake potentially through the ABI5-MYB-bHLHs-IRT1 and ABI5-MYB49-HIPPs pathways in Arabidopsis (**Figure 1A**), and the ABA core components including AIT1, ABI1/HAB1/ABI2, and SnRK2.2/2.3 are involved in these processes.

In As stress, Phosphate Transporters (PHTs, PTs) and Nodulin 26-like Intrinsic membrane Proteins (NIPs) are two major families responsible for arsenate (AsV) and arsenite (AsIII) uptake, respectively (Lindsay and Maathuis, 2017; Deng et al., 2020). In Arabidopsis, As(V) uptake is mainly mediated *via* PHT1;1 and PHT1;4 (Shin et al., 2004). An As(V)-responsive transcription factor WRKY6 was identified as a negative regulator for As(V) uptake through directly repressing expression of *PHT1;1* and removal of PHT1;1 from the plasma membrane (**Figure 1B**) (Castrillo et al., 2013). The expression of *WRKY6* was induced by ABA (Song et al., 2016), knockout of *WRKY6* resulted in ABA insensitivity, while *WRKY6*-overexpressing lines showed ABA-hypersensitive phenotypes during seed germination (Huang et al., 2016), indicating that WRKY6 functions as a positive regulator in ABA signaling. Therefore, we speculate that ABA may inhibit As(V) uptake through WRKY6-PHT1;1 route in Arabidopsis, which requires future investigation. It's also worthy to isolate and functionally characterize rice and brake fern homologs of AtWRKY6 in As(V) uptake because OsPT1, OsPT4, OsPT8, PvPHT1, PvPHT1;3, and PvPHT1;4 confer to As(V) accumulation and toxicity in rice and *P. vittata*, respectively, most likely *via* their role in As(V) uptake in roots (Kamiya et al., 2013; DiTusa et al., 2016; Wang et al., 2016; Cao et al., 2017; Ye et al., 2017; Cao et al., 2019; Sun et al., 2019). Arabidopsis NIP1;1, NIP3;1, NIP3;2, and NIP7;1 function in As(III) uptake and accumulation (Lindsay and Maathuis, 2017; Deng et al., 2020), however, the regulation of them being mediated by ABA is still elusive.

Tremendous progress has been made recently in dissecting the entry of Cd and As into plant cells (Clemens and Ma, 2016; Lindsay and Maathuis, 2017; Zhao and Wang, 2020), and the involvement of ABA in Cd and As(V) uptake is strongly supported by experimental evidence. By contrast, the molecular understanding of Pb uptake pathways is limited. In Pb stress, the plasma membrane-localized G-type ABC members AtABCG36 (PDR8, for Cd and Pb), AtABCG40 (PDR12, for Pb) function as extrusion pumps conferring Pb resistance by limiting their accumulation in Arabidopsis (Lee et al., 2005; Kim et al., 2007; Fu et al., 2019; Wang et al., 2019). Most recently, it has been reported that the expression of *PcABCG40* were stimulated by exogenous ABA in Pb-exposed Gray Poplar (Shi et al., 2019), while the transcription of *AtABCG40* is activated by Pb-Sensitive 1 (AtPSE1), which is a cytoplasmic protein conferring to Pb tolerance in Arabidopsis (Fan et al., 2016). Besides, Arabidopsis *ABCG36* is positively regulated by WRKY13 through directly binding to its promoter (Sheng et al., 2019). Therefore, further studies are required for uncovering the involvement of ABA in Pb uptake, such as the role of ABA in Pb extrusion from root, potentially *via* ABCGs, PSE1, and WRKY13.

## ABA Alters Toxic Metals and Metalloids Distribution Between Root and Shoot

Exogenous ABA tends to hinder metal ion and metalloid translocation to the shoot *via* inhibiting transpiration. Xylem loading is a limiting step for metal ion and metalloid accumulation in the above-ground tissues; the activity is largely dependent on transpiration activity and membrane transporters (Uraguchi et al., 2009; Clemens and Ma, 2016; Deng et al., 2018; Deng et al., 2019). For Cd stress, pre-treatment with ABA dramatically reduced Cd accumulation in the leaves of Indian mustard (Salt et al., 1995) and less Cd accumulation was detected in parallel with a reduced transpiration rate and stomatal closure with up to 100 μM ABA treatment in rice and *Arabidopsis helleri* (Salt et al., 1995; Hsu and Kao, 2003; Zhao et al., 2006; Uraguchi et al., 2009). Besides, involvement of transpiration capacity in the ABA-induced reduction of Cd allocation from roots to leaves is yet to be validated in lettuce (*Lactuca sativa*) and field mustard (*Brassica campestris*) (Shen et al., 2017; Tang et al., 2020).

Membrane transporters play critical roles in loading minerals toward root vascular tissues and subsequent accumulation in plants (Song et al., 2014a; Clemens and Ma, 2016; Deng et al., 2019; Zhao and Wang, 2020). For example, the plasma membrane-localized heavy metal ATPases (HMAs), such as AtHMA2, AtHMA4, and OsHMA2, confer to loading Cd into the stele and subsequent accumulation in shoot (Hussain et al., 2004; Mills et al., 2005; Yamaji et al., 2013), while the tonoplast-localized AtHMA3 and OsHMA3 limit Cd allocation to the stem, leaves, and grain through sequestration Cd into the vacuole of root cells (Morel et al., 2009; Ueno et al., 2010). As a result, the mutant plants without functional *AtHMA2*, *AtHMA4*, or *OsHMA2* accumulate less Cd compared to the wild type (Hussain et al., 2004; Mills et al., 2005; Yamaji et al., 2013). Knockout of *AtHMA3* or *OsHMA3* increased Cd accumulation in aerial organs (Morel et al., 2009; Ueno et al., 2010), whereas overexpressing *OsHMA3* enhanced Cd tolerance and produced Cd-free rice lines (Ueno et al., 2010; Sasaki et al., 2014; Lu C. et al., 2019). Similar functions of their homologs have been reported in other plants, including barley (*Hordeum vulgare*), wheat (*Triticum aestivum*), soybean (*Glycine max*), cucumber (*Cucumis sativus*), and Cd hyperaccumulators *Sedum plumbizincicola*, *Noccaea caerulescens*, and *Arabidopsis halleri* (Hanikenne et al., 2008; Miyadate et al., 2011; Ueno et al., 2011; Mills et al., 2012; Wang et al., 2012; Tan et al., 2013; Migocka et al., 2015; Liu H. et al., 2017). Natural variations in the *HMA3* genes are key determinants of Cd translocation to and accumulation in the shoot of Arabidopsis, rice, soybean, and *Brassica rapa* (Chao et al., 2012; Wang et al., 2012; Liu et al., 2019; Sui et al., 2019; Zhang L. et al., 2019). In addition, some members belonging to NRAMPs also transport free Cd ions (Clemens and Ma, 2016; Zhang et al., 2018). For instance, the expression of *HMA3*, *Nramp1*, *Nramp3*, and *Nramp4* were upregulated in Arabidopsis incubation with ABA-catabolizing bacteria (Lu et al., 2020a), but overexpression of *MhNCED3* in Arabidopsis inhibited the expression of *IRT1*, *Nramp1*, and *HMA2*, leading to reduced Cd uptake and root-to-shoot translocation (Zhang W. et al., 2019). Application of ABA promotes Cd resistance and mobility from root to shoot in the Cd-hyperaccumulating ecotype (HE) of *Sedum alfredii* by inducing the transcription of *SaHMA2*, *SaHMA3*, and *SaHMA4* (Lu et al., 2020b). Interestingly, more endogenous ABA was generated in the non-hyperaccumulating ecotype (NHE) subjected to Cd treatment and induced the expression of lignin- and suberin-related biosynthetic enzymes in NHE roots to limit Cd radial transport towards the stele as well as accumulation in the shoot (Tao et al., 2019). Moreover, some membrane transporters are indirectly involved in ABA-related Cd tolerance. In Arabidopsis, nitrate transporter NRT1.5 plays a vital role in the root-to-shoot translocation of nitrate (Lin et al., 2008), while NRT1.8 is responsible for removing nitrate from xylem vessels and also confers tolerance to CD in a nitrate-dependent manner (Li et al., 2010). Exogenous ABA inhibits the expression of *NRT1.5* but has no effect on the transcripts of *NRT1.8*, leading to increased accumulation of nitrate in the roots and thus enhances Cd resistance (Wang et al., 2018). Vacuolar proton pumps V-ATPase and V-PPase are able to resist Cd through enhanced compartmentation activity into root vacuoles (Wang et al., 2018). Knockout of *BGLU10* or *BGLU18* reduced endogenous active ABA level, resulting in higher levels of *NRT1.5* and lower V-ATPase and V-PPase activities, resulting in higher Cd accumulation and sensitivity (Wang et al., 2018). However, the direct regulators of these transporters response to ABA remains to be discovered and functionally characterized.

**Figure 2:**
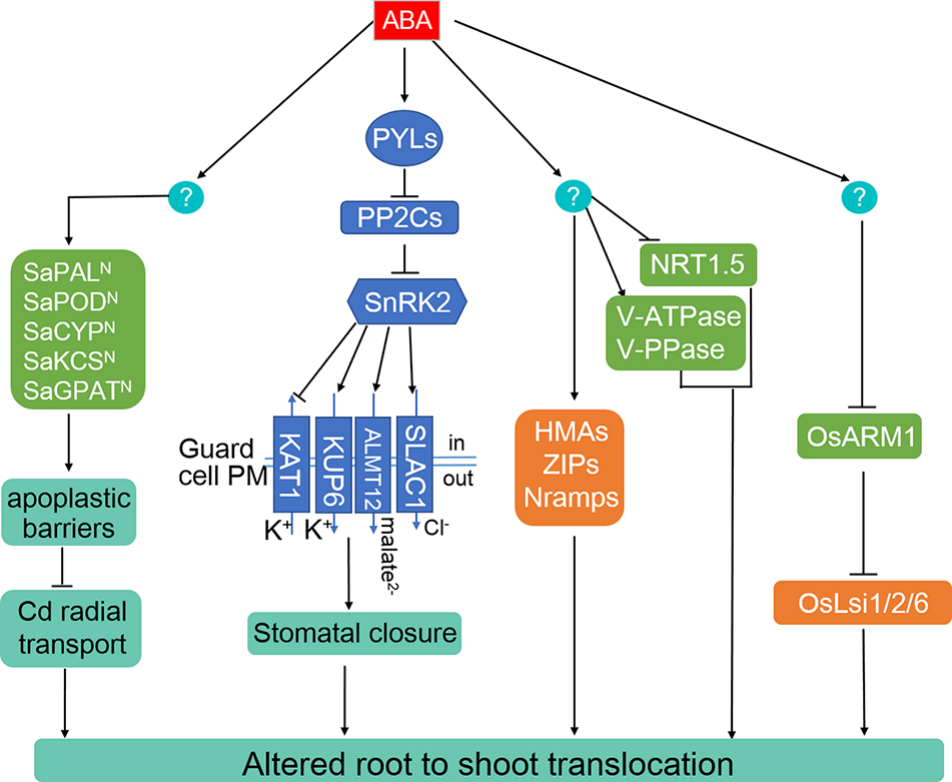
ABA alters the root-to-shoot translocation of Cd, As, and Pb through multiple routes. ABA is able to promote the generation of root apoplastic barriers to inhibit Cd radial transport toward stele in the non-hyperaccumulating ecotype of *Sedum alfredii*. ABA-induced stomatal closure repress the allocation of Cd, As, and Pb from root to shoots. ABA can change the distribution of heavy metals by inducing the expression of certain *HMAs*, *ZIPs*, and *Nramps* transporter genes in various plant species. ABA change Cd distribution between root and shoots through increasing the activates of vacuolar protons, V-ATPase, and V-PPase and inhibiting nitrate movement from root to shoot mediated by NRT1.5. In rice, As uptake, distribution, and tolerance mediated by OsLsi1, OsLsi2, and OsLsi6 is repressed by the ABA-inhibited transcriptional factor, OsARM1.

For As stress tolerance and translocation, many of the fundamental studies were conducted in rice. For instance, a plasma membrane-localized ABC transporter OsABCC7, highly expressed in the root xylem parenchyma cells, is involved in the root-to-shoot translocation of glutathione (GSH)- and Phytochelatins (PCs)-conjugated As (Tang Z. et al., 2019). As(III) uptake and loading to shoot are predominantly accomplished by plasma membrane-polar localized OsLsi1 and OsLsi2 in rice (Ma et al., 2008). OsLsi1 is a member of NIPs required for As(III) uptake from soil into the root cells while OsLsi2 is responsible for the subsequent As(III) transport out of epidermal and endodermal cells toward the stele (Ma et al., 2008). Knockout of *OsLsi1* reduced As uptake, while mutation of *OsLsi2* decreased As accumulation in rice straw and grain (Ma et al., 2008). OsLsi6, a homolog of OsLsi1 highly expressed in rice nodes, was implicated as a transporter required for As distribution from leaf and node to panicle (Yamaji et al., 2015; Deng et al., 2020). The expression levels of *OsLsi1*, *OsLsi2*, and *OsLsi6* were negatively regulated by Arsenite-Responsive MYB 1 (OsARM1) through the direct binding to the promoters or genomic regions of the three key As transporters (**Figure 2**) (Wang F.-Z. et al., 2017). Knockout of *OsARM1* improved tolerance to As(III) and increased As accumulation in shoot and the upmost node, while As concentrations in these organs and the tolerance to As(III) were reduced in *OsARM1-*overexpressing plants compared to those of wild-type plants (Wang F.-Z. et al., 2017). Most interestingly, it was found that the expression of *OsARM1* was repressed by exogenous ABA treatment (Sato et al., 2011; Guo et al., 2016). Therefore, we propose that ABA may enhance As tolerance by promoting As accumulation in above-ground tissues, which is partially dependent on the OsARM1-OsLsi1/Lsi2/Lsi6 pathway. The direct components for the ABA-inhibited transcription of *OsARM1* require further investigations.

For Pb stress tolerance, stomatal closure and a decreased transpiration ratio were observed in the pea leaves with Pb exposure, accompanied with elevated amount of endogenous ABA (Parys et al., 1998). Moreover, the mobility of Pb from root to shoot in rice and Gray Poplar (*Populus × canescens*) was also affected by exogenous ABA in a dose-dependent manner. When subjected to 10 μM exogenous ABA, stimulated Pb uptake and vascular loading in the roots was observed in Gray Poplar (Shi et al., 2019). In rice, a low concentration of ABA (0.38 μM) decreased Pb translocation from roots to shoots, whereas a high concentration of ABA (3.8 and 38 μM) resulted in antipodal Pb distribution (Zhao et al., 2009). However, the genetic evidence underlying the ABA-affected Pb distribution is still lacking.

In summary, ABA is important in regulating the root-to-shoot translocation ratio of heavy metals and metalloids. Many physiological investigations suggest that ABA-induced stomatal closure and a reduced transpiration rate limit the long-distance transport of heavy metals and metalloids from root to shoot. Numerous transporters for Cd, As, and Pb distribution have been identified in Arabidopsis and rice, potentially supporting the participation of ABA in these processes.

## ABA Promotes Chelation and Vacuolar Sequestration of Toxic Metals and Metalloids

The cytosolic Cd, As, and Pb can be conjugated by thiol-containing chelators, GSH, and/or PCs and then compartmented into vacuoles. These processes play pivotal roles in the detoxification of toxic metals and metalloids by restricting their mobility (Clemens et al., 1999; Gong et al., 2003; Kim et al., 2006; Song et al., 2010; Park et al., 2012; Song et al., 2014b; Hayashi et al., 2017; Deng et al., 2018; Deng et al., 2019). It is well documented that GSH is synthesized through γ-glutamylcysteine synthetase (GSH1 or γ-ECS) and glutathione synthetase (GSH2), while PCs are polymerized from GSH by PCS (PCS1, PCS2) in Arabidopsis (Cobbett, 1999; Vatamaniuk et al., 1999; Cobbett, 2000).

**Figure 3:**
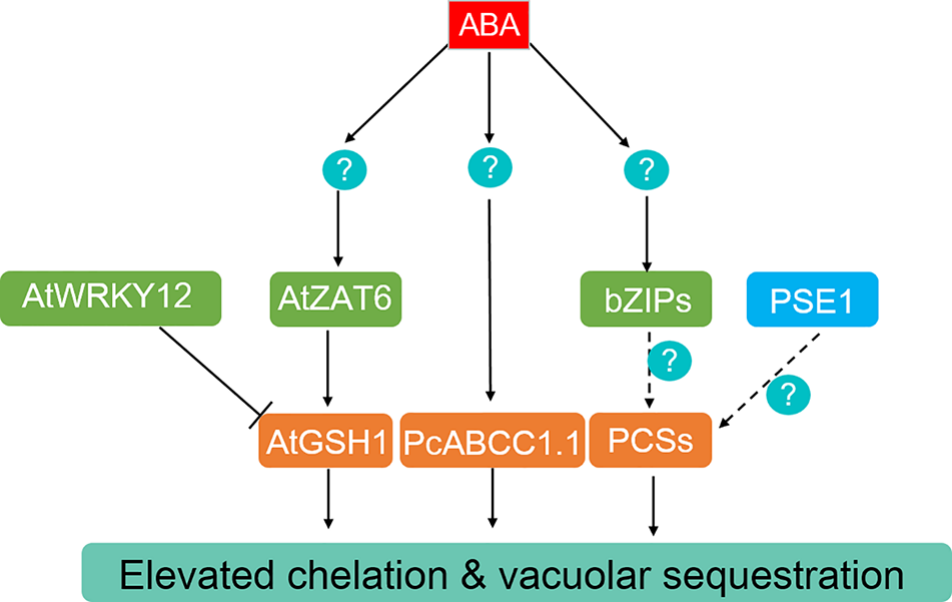
ABA-dependent and -independent regulation of heavy metals and metalloids chelation and compartmentation. The generation of GSH and PCs can be promoted by the ABA-induced transcriptional factors AtZAT6 in Arabidopsis, bZIP members in different plant species. AtWRKY12 functions as an ABA-independent negative regulator of AtGSH1, while PCS expression is enhanced by PSE1. Moreover, PcABCC1.1 is activated by ABA and may participate in Pb sequestration in Gray Polar.

In Arabidopsis, the transcription of *GSH1*, *GSH2*, *PCS1*, and *PCS2* are positively regulated by a Cd-induced Cysteine-2/Histidine-2 type zinc-finger (C_2_H_2_) transcriptional factor, ZAT6 (Chen J. et al., 2016). Overexpressing *ZAT6* significantly enhanced Cd tolerance, whereas loss of function of *ZAT6* led to Cd sensitivity (Chen J. et al., 2016). In a systematic study of the ABA transcriptional regulatory network, the expression of *ZAT6* was upregulated by exogenous ABA treatment (Song et al., 2016). In addition to activating the transcription of *ABCG40*, genes involved in PCs synthesis are also activated by PSE1, which confers Pb tolerance in Arabidopsis (**Figure 3**) (Fan et al., 2016). WRKY12 negatively regulates Cd tolerance by repressing the expression of PCs synthesis genes (Han et al., 2019). Recently, OsPCS1 and OsPCS2 were identified and played crucial roles in the detoxification and accumulation of As and Cd in rice (Hayashi et al., 2017; Uraguchi et al., 2017; Yamazaki et al., 2018). Loss-of-function of *OsPCS1* increased As allocation from node to seed, while overexpressing *OsPCS1* significantly reduced grain As content (Hayashi et al., 2017). Overexpression of wheat TaPCS1, *Morus notabilis MnPCS1*, and *MnPCS2* or *Populus tomentosa PtPCS* in Arabidopsis and/or tobacco enhanced Cd tolerance (**Figure 3**) (Fan et al., 2018). In addition, a plastid envelope membrane-localized CRT-like transporter, OsCTL1, is required for As and Cd detoxification through exporting γ-glutamylcysteine and GSH from plastids to the cytoplasm, where PCS synthesis takes place (Yang et al., 2016). Treatment of potato (*Solanum tuberosum*) plants with ABA clearly enhanced *StPCS1* transcript level, PCS activity, and PCs content in roots, while application of the ABA biosynthesis inhibitor, fluridone, limited the Cd-induced PCS activity (Stroiński et al., 2010; Stroiński et al., 2013). ABA-induced expression of *StPCS1* was in parallel with an elevated level of *StbZIP*, encoding a potential ABF on the upstream of *StPCS1* (**Figure 3**) (Stroiński et al., 2010; Stroiński et al., 2013). Similarly, the transcript levels of *PcECS1* and *PcPCS1.1* genes, encoding rate-limiting enzymes for GSH and PCs synthesis, were upregulated in the roots of Gray Poplar treated with ABA compared with the control, irrespective of Pb treatments (**Figure 3**) (Shi et al., 2019).

The members of the ABC transporter family mediate the transport of GSH- or PCs-conjugated toxic metals and metalloids for detoxification (Song et al., 2010; Park et al., 2012; Song et al., 2014b; Brunetti et al., 2015). The vacuolar membrane-localized Arabidopsis ABCC1 and ABCC2 mediate tolerance to both Cd and As by sequestrating the complexes into vacuoles, while ABCC3 only confers Cd detoxification (Song et al., 2010; Park et al., 2012; Brunetti et al., 2015). Knockout of *OsABCC1* resulted in enhanced As sensitivity and largely increased As accumulation in rice grain, and ectopic expression of *OsABCC1*, *ScYCF1* (*Saccharomyces cerevisiae yeast cadmium factor 1*, homolog of ABCC1 in yeast), and *γ-ECS* reduced grain As content by 70% compared to that of control by trapping AS-PCs into vacuoles of root cortical cells (Song et al., 2014b; Deng et al., 2018), indicating the critical role of OsABCC1 in As tolerance and reducing As distribution to grains. Overexpression of *ScYCF1* in Arabidopsis enhanced tolerance and accumulation of Cd and Pb (Song et al., 2003). The mitochondrial transporter ABCB25 (ATM3) contributes to Cd and Pb resistance, possibly by transporting glutamine synthetase (GS)-conjugated complexes across the mitochondrial membrane (Kim et al., 2006). In addition to the upregulated expression of genes for PCs synthesis, the mRNA levels of the *PcABCC1.1* was increased by exogenous ABA in Pb-exposed Gray Poplar (**Figure 3**) (Shi et al., 2019), indicating a possible enhanced Pb compartmentation activity of plants with ABA treatment.

Taken together, ABA alleviates heavy metals and metalloids' toxicity partially through increasing the generation of GSH and PCs as well as the vacuolar sequestration capacity of the conjugated complexes. PSE1 and transcriptional factors bZIPs, C_2_H_2,_ and WRKYs are the likely candidates responsible for the ABA-induced GSH and PCs production. However, the evolutionary origin and conservation of this regulatory pattern in land plants and algae needs further evidence.

## Evolution of Gene Families for ABA-Responsive Heavy Metals and Metalloids Detoxification

Growing evidence has revealed the evolutionarily conserved roles of ABA and its biosynthesis and signaling systems from bryophytes to angiosperms that cause them to survive and thrive in terrestrial conditions (Sakata et al., 2014; Cuming and Stevenson, 2015; Shinozawa et al., 2019). However, our understating of ABA signaling processes leading to heavy metals detoxification in non-angiosperms is still limited. Using a range of available tools in bioinformatics and plant evolutionary biology (Leebens-Mack et al., 2019; Zhao et al., 2019; Adem et al., 2020), we were able to glimpse the evolution of gene families for ABA-responsive heavy metals and metalloids' detoxification and trace the origin and co-evolution of ABA signaling and tolerance to metals and metalloids in plants.

### ABA Biosynthesis and Signaling Network

**Figure 4:**
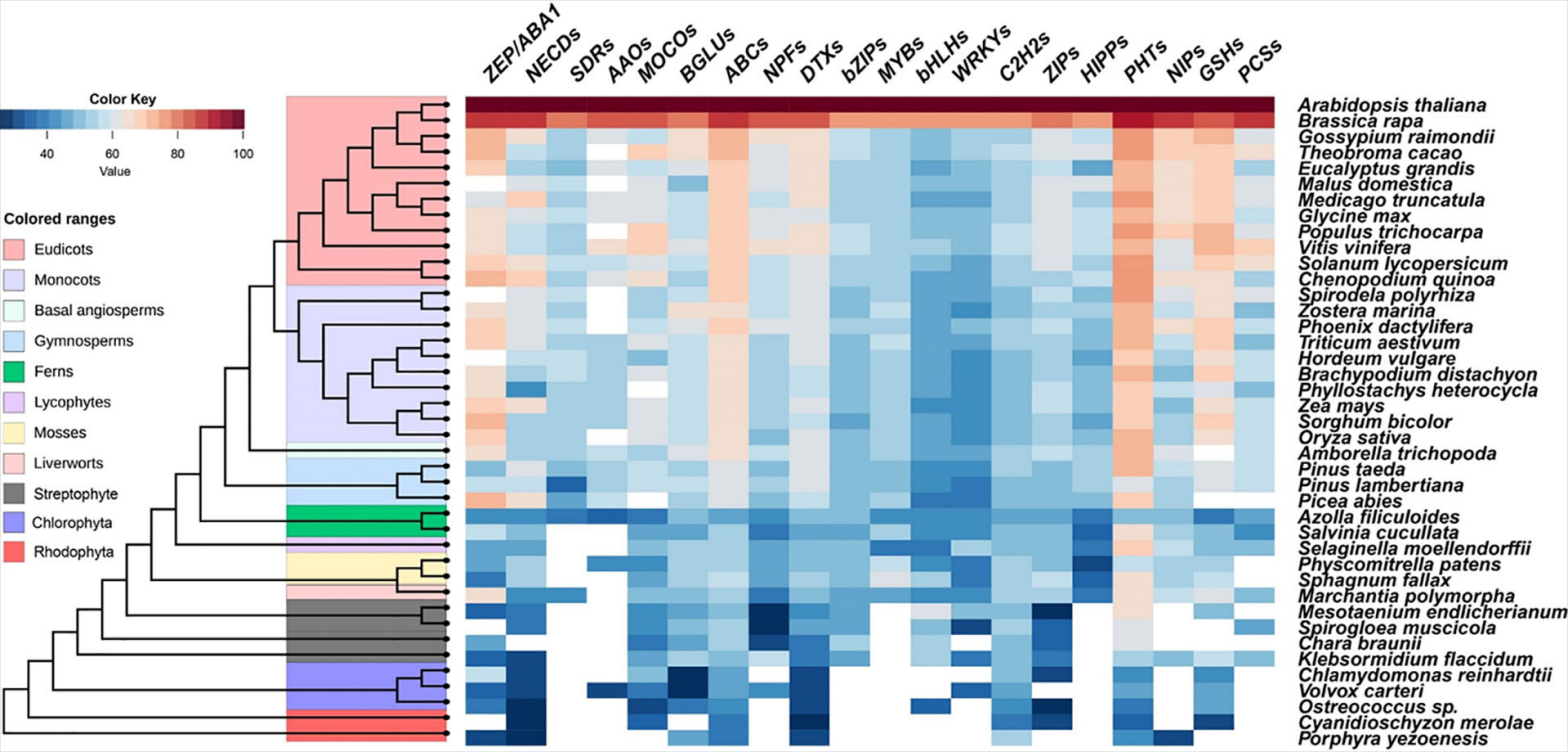
Similarity heat map of ABA signaling components and ABA-responsive heavy metals and metalloids-related proteins in different species. Candidate protein sequences were selected by BLASTP searches which satisfied E value <10^-10^ and query coverage >50%. Colored squares indicate protein sequence similarity from zero (Blue) to 100% (red). White squares indicate proteins that satisfied neither of the selection. ZEP, zeaxanthin epoxidase; NCEDs, 9-cis-epoxycarotenoid dioxygenase; SDRs, short-chain alcohol dehydrogenase/reductases; AAOs, abscisic aldehyde oxidases; MOCOs, molybdenum cofactor sulfurase; BGLUs, β-glucosidases; ABCs, ATP-binding cassette transporters; NPFs, Nitrate Transporter 1/Peptide Transporter; DTXs, DTX/Multidrug and Toxic Compound Extrusion (MATE); bZIPs, Basic region/leucine zipper proteins; MYBs, MYB transcriptional factors; bHLHs, basic helix-loop-helix transcriptional factors; WRKYs, WRKY transcriptional factors; C_2_H_2_s, Cysteine-2/Histidine-2 type zinc-finger transcriptional factors; ZIPs, Zinc Regulated Transporter/Iron Regulated Transporter-like Proteins; HIPPs, Heavy metal-associated Isoprenylated Plant Proteins; PHTs, Phosphate transporters; NIPs, Nodulin 26-like Intrinsic membrane Proteins; GSHs, glutathione synthetases; PCSs, Phytochelatin synthetases.

As described above, physiological and genetic evidence reveals the involvement of ABA in plants resistance to Cd, As, and Pb stresses. Certain members belonging to ZEPs, NCEDs, AAOs, MOCOs, BGLUs, AITs (NPF4s), SnRK2s, and ABFs (bZIPs) are positive regulators, while PP2Cs play negative roles. Bioinformatics analyses were performed to identify the predicted gene families responsible for ABA biosynthesis, catabolism, transport, signal perception, and transduction in these 10 gene families across 41 species, including chlorophyte and streptophyte algae, red algae, and plants (**Figure 4**). All ZEPs, NCEDs, MOCOs, BGLUs, ABC transporters, and DTX transporters have been identified across most tested land plant and algal species (**Figure 4**), which showed the same pattern to those of SnRK and PP2C protein families (Cai et al., 2017; Chen et al., 2017). The orthologs of SDRs and AAOs were mainly identified in fern *Azolla filiculoides* and seed plants but not in any algae. NPFs were found in Chlorophyta *Volvox carteri*, all tested Streptophyte, and land plants, but not in red algae (**Figure 4**). Molecular and genetic evidence revealed that core ABA signaling networkz consisting of PYR/PYL/RCARs, PP2Cs, and SnRK2s of early land plants is comparable to that of Arabidopsis (Tougane et al., 2010; Takezawa et al., 2015; Bowman et al., 2017; Briskine et al., 2017; Cai et al., 2017; Eklund et al., 2018; Jahan et al., 2019; Shinozawa et al., 2019). As a result, the ABA signaling network evolved before the land plants.

The above summarized experimental evidence revealed that ABFs belonging to bZIPs are the main regulators for Cd, As, and Pb uptake, distribution, and detoxification regulated by ABA signaling. The origin of land plant gene families that are relevant to ABA and metal and metalloid tolerance can be traced to Streptophyte algae with 7 members of bZIP transcriptional factors in the basal Streptophyta species *Klebsormidium flaccidum* and *Chara braunii* (**Figure 4**) (Cai et al., 2017; Chen et al., 2017; Zhao et al., 2019; Adem et al., 2020), indicating an early evolution of these gene families in Viridiplantae (green plants). The *bZIPs* gene family has since evolved and diversified into multiple members based on genome assembly of recently sequenced Streptophyte algae *Spirogloea muscicola* (**Figure 4**) in the Zygnematophyceae family (Cheng et al., 2019), which includes mosses, liverworts, lycophytes, ferns, gymnosperms, and angiosperms. For instance, there are 78 genes in the *bZIP* family in *Arabidopsis thaliana* (Jakoby et al., 2002) and the evolution of orthologues of *bZIPs* can be identified in the basal lineage of mosses: *Sphagnum fallax* in Sphagnopsida and *Physcomitrella patens* in Bryopsida (**Figure 4**).

Regulation of stomata and transpiration are vital for plants' tolerance to heavy metals and metalloids by reducing their uptake. Rapid stomatal responsiveness to fluctuant environmental stimulus is an essential adaptation to terrestrial plant life (Cai et al., 2017; Chen et al., 2017; Zhao et al., 2019). The opening and closing of stomata is predominantly dependent on ABA, which can be induced in plants under heavy metals and metalloids' stress (Amir et al., 2018; Chen et al., 2020). In flowering plants, stomata are directly regulated by ion flux mediated by several guard cell membrane-localized transporters, which are triggered by ABA in the Ca^2+^-dependent and Ca^2+^-independent pathway (Cai et al., 2017; Chen et al., 2017; Chen et al., 2019; Zhao et al., 2019; Chen et al., 2020). Comparative genomics and transcriptomics revealed that the ABA reception complex protein families including RCARs, PP2Cs, and SnRK2s, guard cell transporter families consisting of SLACs, KATs, and ALMTs, and kinases CDPKs and CIPKs critical for ABA-induced stomatal closure have been identified across the land plant species with stomata (Lind et al., 2015; Chater et al., 2016; Cai et al., 2017; Chen et al., 2017; Zhao et al., 2019). The physiological roles of certain members from liverworts, mosses, and ferns have been verified through genetic complementation tests (Tougane et al., 2010; Chater et al., 2011; Ruszala et al., 2011; Komatsu et al., 2013; Lind et al., 2015; Cai et al., 2017). The results reveal that the molecular mechanism underlying ABA-regulated stomatal aperture tends to be similar across plant lineages, but the stomata of vascular plants are generally more responsive to ABA than those in early plant species (Chen et al., 2017). In addition to ABA-regulated stomata responsiveness, the emergence of functional root and vascular tissues, which connect the various organs of plants and are essential for the long-distance transport of minerals, is indispensable for the Cd, As, and Pb distribution among tissues driven by ABA. Therefore, we propose that ABA is able to regulate heavy metals and metalloids' distribution in all vascular plant species, which of course remain to be investigated by the plant research community.

### Regulation of ABA-Responsive Heavy Metals and Metalloids Detoxification

Some proteins responsible for the uptake, distribution, chelation, and compartmentation of Cd, As, and Pb are directly or indirectly regulated by ABA. Here, we identified the orthologues of transporter families including ZIPs, HIPPs, PHTs, NIPs, NPFs, and ABCs, transcriptional factor families consisting of bZIPs, MYBs, bHLHs, WRKYs, and C_2_H_2_s, enzymes for GSH and PCs synthesis from 41 plant and algal genomes (**Figure 4**).

In Arabidopsis, ABA is able to inhibit Cd uptake and accumulation mediated by IRT1, HIPP22, and HIPP44 at transcriptional levels (Zhang P. et al., 2019), while reducing endogenous ABA content increased the expression of *IRT1* and its homologs, *ZIP1* and *ZIP4* (Lu et al., 2020a). IRT1 is a member of the ZIP family with board substrates including iron (Fe), zinc (Zn), cobalt, manganese (Mn), and Cd (Rogers et al., 2000; Vert et al., 2002). Homologs of ZIPs were identified in almost all examined algae and land plants, except in Rhodophyta *Porphyra yezoenesis* and Chlodophyta *Volvox carteri* (**Figure 4**), indicating the early arising of ZIPs prior to the evolution of land plants. There are 17 ZIPs in Arabidopsis, and IRT1-like members are only found in angiosperms (Lo et al., 2016). For example, five and four ZIPs were isolated from *M. polymorpha* and *P. patens* respectively; they were clustered into an IRT3-like group encoding Fe/Zn transporters in Arabidopsis and ZIP2-like subgroup transporting Zn, Fe, and Mn but not Cd (Lo et al., 2016). The activation of *IRT1* by MYB49 requires Ib subgroup members of bHLHs to act as the bridge regulators, while the expression of *HIPP22* and *HIPP44* are directly regulated by MYB49 (Zhang P. et al., 2019). The origin of bHLHs can be traced to Chlorophyta, but MYBs and HIPPs appear to be land plant specific (**Figure 4**). Therefore, the analysis implicated that the ABI5-MYB49-bHLH-IRT1 pathway is likely to be conserved in land plants.

Arsenate uptake mediated by PHT1;1 can be restricted by WRKY6 transcriptional factor in Arabidopsis (Castrillo et al., 2013), while As(III) take up and distribution mediated by NIPs can be repressed by MYB protein OsARM1, whose expression is downregulated by ABA (Wang F.-Z. et al., 2017). Both As(V) and phosphate (P), which is an essential macro element for all organisms, are the substrates of PHTs (Lindsay and Maathuis, 2017). Four, eight, and twelve PHT orthologues are identified in *Porphyra yezoenesis*, Chlorophyta *Ostreococcus* sp., and *Klebsormidium flaccidum*, but the numbers are rapidly expanded to 25 in moss *Physcomitrella patens*, 26 in rice, and 19 in Arabidopsis (**Figure 4**). In addition, the PHT proteins show high similarity (over 60%) among all the land plants (**Figure 4**). Transcription factors WRKYs tend to be widespread in all the species examined, except Rhodophyta (**Figure 4**). The putative NIPs are found in the most ancient Rhodophyta *Porphyra yezoenesis*, basal Streptophyta *Klebsormidium flaccidum*, and all land plants examined (**Figure 4**). The origin of NIPs is found from horizontal gene transfer of bacterial aquaporin group with As efflux activity, and NIPs from Charophytes, mosses, and angiosperms are permeable to As (Pommerrenig et al., 2020). Moreover, Arabidopsis NPFs are divided into 8 subgroups (Léran et al., 2014), 4 NPF4s are ABA-importing transporters (Pan et al., 2020), while 2 NPF7s indirectly participate in ABA-altered Cd distribution in roots and shoots (Wang et al., 2018). Using 53 Arabidopsis NPFs as reference, we identified 1,990 orthologs from 36 representative genomes consisting of Chlorophyta *Volvox carteri*, Streptophyte algae, and land plants (**Figure 4**). Among the 2,398 putative NPFs identified from 33 genomes, 350 members were clustered into NPF4s and 206 belonged to NPF7s (Léran et al., 2014), which may be the candidates with ABA transport activity and tolerance to Cd induced by ABA in different plant species, respectively.

The ubiquitous thiol-containing small peptide, PCs, protect cells against the toxic effects of heavy metals and metalloids (Clemens, 2006) and the synthesis of PCs is catalyzed by PCS using reduced GSH and related thiols as substrates (Cobbett, 1999). Genes encoding GSHs can be traced to an ancestral streptophyte alga *Klebsormidium flaccidum* (also found in *Cyanidioschyzon merolae*, an unicellular red alga the phylum Rhodophyta), and the similarity among the members from monocots and dicots is over 60% (**Figure 4**). The potential PCS orthologs are found in the *Klebsormidium flaccidum*, *Spirodela polyrhiza*, liverwort *Marchantia polymorpha*, and most vascular plants. However, the activities and responsiveness to various metals of PCS are divergent in various kinds of plants. In general, the PCSs of basal plants appear to be less active compared with Arabidopsis PCS (Degola et al., 2014; Petraglia et al., 2014). Expression of *GSHs* and *PCSs* could be activated by ABA-induced C_2_H_2_ transcriptional factor AtZAT6 and StbZIP but repressed by AtWRKY12 (Stroiński et al., 2013; Chen J. et al., 2016; Han et al., 2019). We found that the C_2_H_2_ member family shows the same evolutionary origin as that of GSHs, whereas WRKYs may be originated from Chlorophyta (**Figure 4**). Transcriptional factors belonging to the bZIP subfamily activates *PCS* transcription in potato and Gray Poplar subjected to exogenous ABA (Stroiński et al., 2010; Stroiński et al., 2013; Shi et al., 2019), which is consistent with the parallel evolution of bZIPs and PCSs from Streptophyte algae (**Figure 4**). Further experiments have to be conducted to test the conservation of these regulatory modules in different plant species.

ABC transporters are important for ABA signaling and responses to heavy metals and metalloids. For instance, ABCB25 has a role in Cd and Pb resistance in Arabidopsis and C-type ABC transporters are involved in sequestration of GSH- and PC-conjugated heavy metals and metalloids in various angiosperms. G-type ABC transporters contribute to ABA transport as well as Cd and Pb efflux (Song et al., 2014a; Hwang et al., 2016). We found that ABC transporters are ubiquitous in all 41 genomes used for comparative genetic analysis (**Figure 4**). There are 69, 125, 130, and 133 ABC transporters in green algae *Chlamydomonas reinhardtii*, moss *Physcomitrella patens*, Arabidopsis, and rice, respectively (Hwang et al., 2016). In Rhodophyta *Porphyra yezoenesis*, 9, 3, and 2 ABC transporter homologs are classified into B-, C-, and G-type subgroups, respectively. The numbers of B-type, C-type, and G-type members increased substantially to 20, 14, and 42, in *Physcomitrella paten* and 30, 17, and 43 and *Arabidopsis*, respectively (Hwang et al., 2016). These are the suggested specific proliferations of ABCB, ABCC, and ABCG subfamily members in land plants. Therefore, it is interesting to verify the evolutionary conservation of the functions of ABC transporters for heavy metals and metalloids' detoxification in the early divergent lineages of plant species.

## Conclusions and Future Perspectives

**Figure 5:**
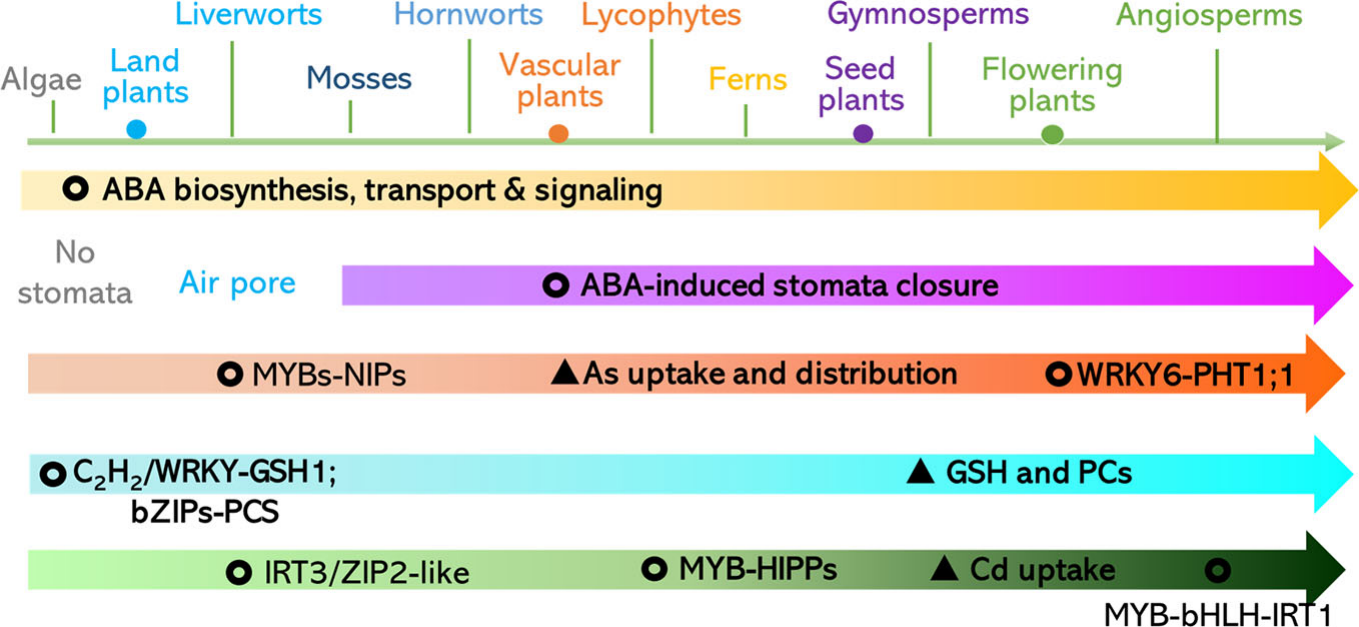
Proposed evolution of ABA-responsive pathways for toxic metals and metalloids uptake, distribution, and detoxification. Please refer to the section of *Conclusions and Future Perspectives* for a detailed description.

In summary, in the key components of the ABA biosynthesis, signaling perception, and transduction pathways, regulatory patterns were evolutionary conserved in land plants but also diversified in different lineages. This may be the case for mechanisms underlying the plant response to toxic metals and metalloids. According to the analyses, we propose that: (1) ABA-repressed Cd uptake mediated by ABI5-MYB49-HIPPs network may be conserved in land plants, (2) ABA-reduced As distribution and detoxification through MYB-NIPs is likely to be conserved in land plants, (3) GSH for metal and metalloid chelation ABA-induced C_2_H_2_ transcriptional factor is likely in green plants, and (4) the earliest origin of bZIP-induced PCS can be traced to the Streptophyta (**Figure 5**). Therefore, future work is suggested to focus on: (1) the discovery of direct regulators of the ABA-responsive transcriptional factors including WRKY6, ZAT6, ARM1, and bZIPs and downstream transporters comprising of ABCs, HMAs, ZIPs, Nramps, NRT1.5, V-ATPase, and V-PPase; (2) characterization of the putative Cd, As, and Pb transporters in major clades of land plants using heterologous expression systems such as yeast and *Xenopus laevis*; and (3) investigation of the roles of heavy metal and metalloid stress-related and ABA-regulated components of major clades of land plants *via* genetic complementation of these genes in corresponding mutants of Arabidopsis and rice. The proposed research will shed light on the practices for mitigation the contaminations. For instance, application of ABA or its analogues in crops for diminishing the accumulation of toxic metals and metalloids and their antagonists can be employed in the hyperaccumulators (e.g. algae, plants) for phytoremediation.

## Author Contributions

Z-HC and FD conceptualized the review. FD, BH, and Z-HC wrote the manuscript with support from XC, WG, LL, JX. GC conducted the comparative genomics analyses and prepared **Figure 4**. FD, BH, and Z-HC did final editing of the manuscript. BH and FD have contributed equally to this work.

## Funding

This work was financially supported by the Engineering Research Center of Ecology and Agricultural Use of Wetland, Ministry of Education and Hubei Key Laboratory of Waterlogging Disaster and Agricultural Use of Wetland (KF201908), and funding from the State Key Laboratory for Conservation and Utilization of Subtropical Agro-bioresources (SKLCUSA-b201910).

## Conflict of Interest

The authors declare that the research was conducted in the absence of any commercial or financial relationships that could be construed as a potential conflict of interest.
